# Cost-Effectiveness of Adding Cetuximab to Platinum-Based Chemotherapy for First-Line Treatment of Recurrent or Metastatic Head and Neck Cancer

**DOI:** 10.1371/journal.pone.0038557

**Published:** 2012-06-20

**Authors:** Malek B. Hannouf, Chander Sehgal, Jeffrey Q. Cao, Joseph D. Mocanu, Eric Winquist, Gregory S. Zaric

**Affiliations:** 1 Department of Epidemiology and Biostatistics, Schulich School of Medicine and Dentistry, University of Western Ontario, London, Ontario, Canada; 2 Richard Ivey School of Business, University of Western Ontario, London, Ontario, Canada; 3 Department of Radiation Oncology, London Regional Cancer Program, London Health Sciences Centre, London, Ontario, Canada; 4 Department of Oncology, Schulich School of Medicine and Dentistry, University of Western Ontario, London, Ontario, Canada; Mahidol-Oxford Tropical Medicine Research Unit, Thailand

## Abstract

**Purpose:**

To assess the cost effectiveness of adding cetuximab to platinum-based chemotherapy in first-line treatment of patients with recurrent or metastatic head and neck squamous cell carcinoma (HNSCC) from the perspective of the Canadian public healthcare system.

**Methods:**

We developed a Markov state transition model to project the lifetime clinical and economic consequences of recurrent or metastatic HNSCC. Transition probabilities were derived from a phase III trial of cetuximab in patients with recurrent or metastatic HNSCC. Cost estimates were obtained from London Health Sciences Centre and the Ontario Case Costing Initiative, and expressed in 2011 CAD. A three year time horizon was used. Future costs and health benefits were discounted at 5%.

**Results:**

In the base case, cetuximab plus platinum-based chemotherapy compared to platinum-based chemotherapy alone led to an increase of 0.093 QALY and an increase in cost of $36,000 per person, resulting in an incremental cost effectiveness ratio (ICER) of $386,000 per QALY gained. The cost effectiveness ratio was most sensitive to the cost per mg of cetuximab and the absolute risk of progression among patients receiving cetuximab.

**Conclusion:**

The addition of cetuximab to standard platinum-based chemotherapy in first-line treatment of patients with recurrent or metastatic HNSCC has an ICER that exceeds $100,000 per QALY gained. Cetuximab can only be economically attractive in this patient population if the cost of cetuximab is substantially reduced or if future research can identify predictive markers to select patients most likely to benefit from the addition of cetuximab to chemotherapy.

## Introduction

There were approximately 4550 new cases of head and neck cancers (excluding thyroid cancer and melanoma) diagnosed in Canada in 2010 [1]. Treatment may include surgery and definitive radiation therapy, with or without concurrent chemotherapy. The main manifestations of treatment failure are loco-regional recurrences and distant metastatic disease. Management of recurrent or metastatic head and neck squamous cell carcinoma (HNSCC) that is inoperable and not amenable to re-irradiation usually involves systemic chemotherapy, with platinum-based combinations being the most commonly used regimens [2]. Regardless of the choice of chemotherapy, this patient population has a poor prognosis with a median survival of six to eight months [3].

Cetuximab (Erbitux) is a chimeric IgG1 monoclonal antibody that competitively inhibits transforming growth factor-α (TGF-α) ligand from binding to epidermal growth factor receptor (EGFR), resulting in inhibition of tumour growth, invasion and metastasis, DNA damage repair and angiogenesis [4], [5], [6].

Cetuximab is the first targeted therapy to demonstrate a significant survival benefit in patients with locally advanced HNSCC [7] and recurrent or metastatic HNSCC [8]. Cetuximab therapy has been recently adopted into clinical practice and funded in most Canadian provinces for patients with locally advanced HNSCC who are platinum-ineligible or elderly because it offers an alternative that is recognized to be superior to radiotherapy alone [9]. A similar adoption strategy has been taken in the United Kingdom [10]. Cetuximab in the recurrent or metastatic HNSCC setting has not yet found its way into clinical practice in Canada [11].

Combined therapy with cetuximab plus platinum-based chemotherapy significantly improved efficacy outcomes compared with platinum-based chemotherapy alone in a randomized phase III trial in patients with recurrent or metastatic HNSCC (the EXTREME study-Erbitux in First-Line Treatment of Recurrent or Metastatic Head and Neck Cancer) [8]. The addition of cetuximab to platinum-based chemotherapy (cisplatin or carboplatin combined with fluorouracil) was associated with a 16% increase in response rate (P<0.001), a 2.3 month increase in progression-free survival (PFS) (P<0.001), and a 2.7 month increase in overall survival (OS) from a median of 7.4 months to 10.1 months (P = 0.036), compared to platinum-based chemotherapy alone [8]. Moreover, the addition of cetuximab to platinum-based chemotherapy did not adversely affect health-related quality of life, as assessed using validated, multidimensional instruments, compared with chemotherapy alone [8]. In the same trial, protocol-defined sub-group analyses indicated that the addition of cetuximab to platinum based chemotherapy is associated with clinical benefits in the majority of the sub-groups investigated and could not demonstrate greater survival benefits to some subgroups than to others [8]. Therefore, the clinical evidence from the EXTREME trial suggests that the combination of cetuximab with platinum-based chemotherapy is the most active first-line treatment regimen currently available for patients with recurrent or metastatic HNSCC and strongly supports the use of this regimen as a standard treatment approach in this patient setting [8]. Recently, the United States Food and Drug Administration (FDA) has approved cetuximab for use in combination with platinum-based chemotherapy for the treatment of recurrent or metastatic HNSCC. The approval was based primarily on the results of the EXTREME trial.

Since the introduction of cisplatin for the treatment of recurrent or metastatic HNSCC approximately 30 years ago, there has been a little improvement in survival among the patients with this disease [12], [13]. Thus, based on the clinical data from the EXTREME trial, cetuximab-based therapy is appealing to both patients and clinicians. According to a recent Canadian analysis, cetuximab costs approximately $6,500 CAD per patient per month with all Canadian health system expenses included [14]. The purpose of this study was to assess the cost-effectiveness of cetuximab plus platinum-based chemotherapy in recurrent or metastatic HNSCC from the perspective of the Canadian public healthcare system.

## Methods

### Model overview

**Figure 1:**
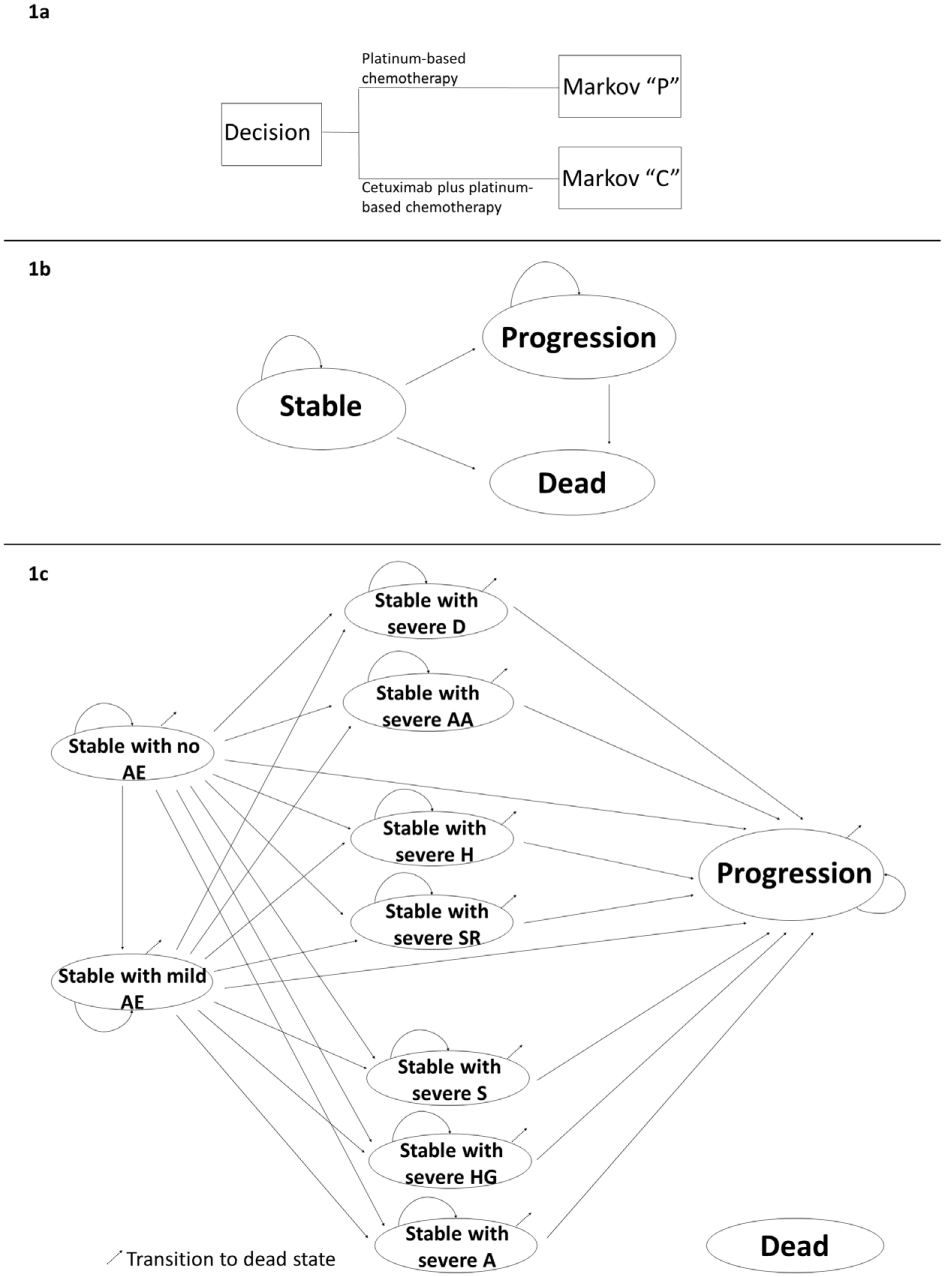
Diagram of the decision model. 1a Decision about choice of treatment regimen. 1b Diagram of Markov model “P”†. 1c Diagram of Markov model “C”‡. Footnotes to Figure 1: †Patients entering Markov model “P” start the model in the stable state and remain in the stable state unless they relapse (progression or death). Patients who progress remain in the progression state or transition to the death state. ‡Patients entering Markov model “C” start the model in the stable state with no AE. During the first cycle patients may develop mild or any severe AE. After the first cycle, patients may remain in stable with no or mild AE unless they develop severe anorexia (A), hypomagnesemia (HG) or sepsis (S), progress or die. Patient who develop any severe AE remain in stable with that AE state unless they progress or die. Patients who progress remain in the progression state or make transition to the dead state. The cycle length was 1 month. AE = cetuximab-related adverse effects, D =  dyspnea, AA =  allergy or anaphylaxis, H =  hypotension, SR =  skin reactions.

We developed a decision analytic model to estimate the health and economic consequences of different treatment regimens for patients with recurrent or metastatic HNSCC (Figure 1; parameter estimates are summarized in Tables 1, 2, 3). The model begins with a decision to treat with cetuximab plus platinum-based chemotherapy or platinum-based chemotherapy alone (Figure 1a). Patients receiving platinum-based chemotherapy entered model “P” (Figure 1b) and those receiving cetuximab plus platinum-based chemotherapy entered model “C” (Figure 1c). Model “C” differs from model “P” in that it has several additional states to account for cetuximab-related adverse effects (AEs). We modeled AEs based on those observed in the EXTREME trial [8] and considered both mild and severe AEs. Mild AEs included grade 1 or 2 infusion-related allergies and skin reactions. Severe AEs included grade 3 or 4 infusion-related reactions (allergy or anaphylaxis, dyspnea and hypotension), anorexia, hypomagnesemia, sepsis and skin reactions.

**Table 1:** Base case probabilities and sources.

Probabilities (per month)			Base Case Value	Duration	Range Tested in Sensitivity Analyses	Distribution used in PSA±	Source
Cetuximab-related adverse events:							
Mild events including infusion-related allergies and skin reactions (grade 1 or 2)			29.2%	First month on cetuximab	25 % – 32.9 %	Beta ( 292, 1000)	[8]
Severe events (grade 3or 4)	Infusion-related reactions	Allergy or anaphylaxis	1.83%	First month on cetuximab	0% – 6%	Beta ( 183, 10000)	[8]
		Dyspnea	0.46%	First month on cetuximab	0%–4.65%	Beta ( 46, 10000)	[8]
		Hypotension	0.46%	First month on cetuximab	0%–4.65%	Beta ( 46, 10000)	[8]
	Skin reactions		8.67%	First month on cetuximab	4.70%–12.7%	Beta ( 867, 10000)	[8]
	Anorexia		0.61%	Time on cetuximab therapy	0%–1.34%	Beta ( 61, 10000)	[8]
	Hypomagnesemia		0.61%	Time on cetuximab therapy	0%–1.34%	Beta ( 61, 10000)	[8]
	Sepsis		0.61%	Time on cetuximab therapy	0%–1.34%	Beta ( 61, 10000)	[8]

± Beta(n, N). Beta distribution was used for other probability parameter estimates not shown in this table.

**Table 2:** Base case utility values and sources.

Health State Utilities			Base Casevalue	Duration	Range Tested in Sensitivity Analyses	Distribution used in PSA±	Source
Stable on platinum-based chemotherapy alone or plus cetuximab (non or mild AE)			0.65	36 months	0.50 – 1.00	Beta ( 650, 1000)	[23]
Progression			0.52	36 months	0.20 – 0.70	Beta( 520, 1000)	[23]
Death state			0				
Utility reductions associated withsevere cetuximab-related adverseevents (grade 3or 4)+	Infusion-related reactions	Allergy or anaphylaxis	−15%	1 month	−25% – −0%	Beta ( 150, 1000)	[26]
		Dyspnea	−36%	1month	−50% – −0%	Beta ( 360, 1000)	[27]
		Hypotension	−8.8%	1 month	−25% – −0%	Beta ( 88, 1000)	[29]
	Skin reactions		−65.7%	2 months	−70% – −0%	Beta ( 657, 1000)	[24]
	Anorexia		− 20%	20 months	−30% – −0%	Beta ( 200, 1000)	[25]
	Hypomagnesemia		−24%	20 months	−30% – −0%	Beta ( 240, 1000)	[30]
	Sepsis		−41%	Life time	−50% – 0%	Beta ( 410, 1000)	[28]

± Beta(n, N).

+ The baseline utility for stable HNSCC (with no or mild AE) was 0.65. We derived the utility for each stable HNSCC state with severe cetuximab-related adverse event (grade 3or 4) by applying utility reduction estimates associated with each severe event to the baseline utility value for stable HNSCC. Thus, the utility of stable HNSCC with a specific severe cetuximab-related adverse event is estimated as 0.65− 0.65 × (utility reduction associated with a severe cetuximab-related adverse event), consistent with methodology described by Fryback et al [31].

**Table 3:** Base case costs and sources.

Costs*(per month), Canadian $					Base Case Value	Duration	Range Tested in Sensitivity Analyses	Distribution used in PSA±	Source
Platinum-based chemotherapy	Chemotherapy acquisition and administration†				635	First 5 months			CCO [15]
	Chemotherapy regimen (cisplatin combined with flurouracil)‡				3,658	First 5 months			LRCP [20]
	Total				4,293	First 5 months	2,000 – 5,000	LogNormal (4,293; 3,850)	
Cetuximab±	Dosing during first month ∥				6,707	First month on cetuximab	-0% – -100%	LogNormal (6,707; 6,300)	PMPRB [18]
	Dosing during followed months∥				5,832	Time on cetuximab therapy following first month	-0% – -100%	LogNormal (5,832; 5,285)	PMPRB [18]
	Infusion time during first month¶				518.2	First month on cetuximab	−0% – −100%	LogNormal (518.2; 470)	[14]
	Infusion time followed months¶				414.5	Time on cetuximab therapy following first month	−0% – −100%	LogNormal (414.5; 380)	[14]
	Pharmacy preparation §				160	Time on cetuximab therapy	−0% – −100%	LogNormal (160; 143)	[14]
Cetuximab-related adverse events (per case)		Mild infusion-related and skin reactions	Consultation fee		143.4	One time			OHIP [49]
			Intravenous antihistamines with cetuximab infusion		804	Time on cetuximab therapy	84–2,516	LogNormal (804; 665)	OCCI [21]
			Combination of hydrocortisone & clindamycin, or minocycline		56	Time on cetuximab therapy	56–88	LogNormal (56; 52)	LRCP [20]
		Severe adverse events	Skin reactions		2,912		335–14,110	LogNormal (2,919; 2,670)	OCCI [21]
			Anorexia		8,436		1,708–18,542	LogNormal (8,436; 7,250)	OCCI [21]
			Hypomagnesemia		5,516		1,658–10,996	LogNormal (5,516; 4,720)	OCCI [21]
			Sepsis		32,462		333–486,612	LogNormal (32,462; 26,860)	OCCI [21]
			Hypotension		3,234		486–15,141	LogNormal (3,234; 2,780)	OCCI [21]
			Allergy or anaphylaxis		3,764		126–21,332	LogNormal (3,764; 3,420)	OCCI [21]
			Dyspnea		3,991		148–33,249	LogNormal (3,991; 3,590)	OCCI [21]
Progression	Inpatient hospice care				25,333	Time with progression	1,230–35,413	LogNormal (25,333; 22,870)	OCCI [21]

* Costs include direct costs and indirect costs. Direct costs are costs that are directly related to the provision of care to the patient and include Nursing (incl. Operating Room, ICU), Diagnostic Imaging, Pharmacy and Labs. Indirect costs are overhead expense relating to the running of hospitals and include administration, finance, human resources, plant operations etc.

± Lognormal(mean, median).

† Chair time: Cancer Care Ontario Drug Formulary [15]; overhead costs: 2002 costs [50] ($ 35/h and $57.42/h respectively) inflated to 2011 using the bank of Canada inflation calculator [51].

‡ Patients receive platinum-based chemotherapy including cisplatin (at a dose of 100 mg/m2 as a 1-hour intravenous infusion on day 1) and an infusion of fluorouracil (at a dose of 1000 mg/m2 per day for 4 days) every 3 weeks for a maximum of 6 cycles; assuming average m2 = 1.8; cisplatin = $448/100 mg; fluorouracil = $147.73 for 100 ml (500 mg vial).

∥ Dosing: 400 mg/m2 initial followed by a weekly infusion of 250 mg/m2; assuming average m2 = 1.8; cetuximab = $3.24/mg;

¶ Infusion time: initial dose infused over 120 min; weekly maintenance dose infused over 60 min; initial dose infusion time/cycle: $103.64/h×2 h = $207.28; maintenance dose infusion time: $103.64/h×1 h = $103.64.

§ Pharmacy preparation time required (e.g. Physician preparation, order processing): Pharmacy preparation time = $40/h×1 h = 40.

CCO = Cancer Care Ontario; LRCP = London Regional Cancer Program; PMPRB: Patented Medicines Prices Review Board; OHIP = Ontario Health Insurance Plan; OCCI: Ontario Case Costing Initiative.

Model “P” simulated monthly transitions among the following distinct health states: (1) Stable (no progression); (2) Progression; (3) Dead. Model “C” simulated monthly transitions among the following distinct health states: (1) Stable with no AE; (2) Stable with mild AE; (3) Stable with severe dyspnea; (4) Stable with severe allergy or anaphylaxis; (5) Stable with severe hypotension; (6) Stable with severe skin reactions; (7) Stable with severe sepsis (8) Stable with severe hypomagnesemia; (9) Stable with severe anorexia; (10) Progression; (11) Dead. We assumed that transitions to any of the stable states with AE except those with severe anorexia, hypomagnesemia and sepsis would only occur in the first month of treatment since these reactions are most likely to start developing following the initial infusion of cetuximab [8]. We assumed that severe anorexia, hypomagnesemia or sepsis could occur any time while the patient still received cetxuximab [8]. We assumed that patients who develop any severe AE or experience progression would stop receiving cetuximab in accordance with Canadian guidelines for the administration of cetuximab [15]. We used a time horizon of 3 years (36 months). This time horizon was appropriate since the overall survival probabilities at 2 years in the EXTREME trial were 18% in the cetuximab plus platinum-based chemotherapy arm and 16% in the platinum-based chemotherapy alone arm, and projected survival beyond 3 years was less than 1% in both groups.

We used TreeAge Software to produce and evaluate the decision analytic model, using a half cycle correction [16].

### Transition probabilities

For both models we derived time-dependent monthly transition probabilities from the “stable” to “progression” state and from the “progression” to “dead” state, respectively, using the Kaplan-Meier curves of progression free survival and overall survival over two years of follow up reported in the EXTREME trial [8]. We used sex-specific life tables for Ontario to adjust the derived transition probabilities of overall survival to account for death by other causes [17]. We assumed that transitions from “stable” to “dead” were from causes other than HNSCC and we estimated these transition probabilities using Ontario sex-specific life tables [17] accounting for the sex balance observed in the EXTREME trial [8]. To extrapolate the transition probabilities for 1 year beyond the period of the EXTREME trial, we assumed the observed average monthly transition probabilities from “stable” to “progression” and from “progression” to “dead” during the second year of follow up in the EXTREME trial to be constant over the extrapolated third year.

We derived the incremental AE rates for cetuximab plus platinum-based chemotherapy versus platinum-based chemotherapy treated patients from the adverse-event profiles provided in the EXTREME trial.

### Cost and Utility Values

In Canada, there is no publicly available source for the cost of cetuximab [18]. The Patented Medicine Prices Review Board (PMPRB) is a government agency in Canada which regulates the prices of drugs that are still under patent and have no generic substitutes. PMPRB guidelines stipulate that the price in Canada cannot exceed the median cost among a set of comparison countries [18]. The cost of cetuximab in 2005 ranged from $2.94 to $6.73 per mg in countries that were reviewed by the PMPRB with a median cost of $3.49 per mg. As of March 2012 cetuximab is reimbursed by Cancer Care Ontario at $3.46 per mg [19]. In our base case analysis we used $3.46 per mg of cetuximab.

The costs of management of mild AEs were obtained from internal case costing conducted by the London Regional Cancer Program, London, Canada [20]. We assumed that any severe AE will result in hospitalization. Hospital costs, based on the Ontario Case Costing Initiative [21], were applied to the corresponding severe AEs using the International Classification of Diseases, tenth revision diagnostic code [22]. All costs are expressed in 2011 CAD.

We assumed that the addition of cetuximab to platinum-based chemotherapy would not adversely affect health related quality of life compared with chemotherapy alone as observed in the EXTREME trial [8]. The baseline utility for stable HNSCC (with no or mild AE) was 0.65 and for progressing HNSCC was 0.52, based on estimates supplied by the manufacturer of cetuximab in its submission to the UK National Institute for Health and Clinical Excellence [23]. To account for the disutility associated with severe AEs, we derived disutility estimates for patients with these events as reported in the literature [24], [25], [26], [27], [28], [29], [30]. We applied these disutility estimates to the baseline utility for stable HNSCC to reflect the utility for stable HNSCC with different severe AEs consistent with methodology described elsewhere [31]. All future costs and utilities were discounted at 5% following Canadian guidelines [32].

## Results

### Base-case scenario

In the base case, the overall survival at 3 years in our model were 0.5% in the cetuximab plus platinum-based chemotherapy arm and 0% in the platinum-based chemotherapy alone arm. Cetuximab plus platinum-based chemotherapy compared to platinum-based chemotherapy alone led to an increase of 0.093 QALY per person and an increase in cost of $36,000 per person, resulting in an incremental cost effectiveness ratio (ICER) of $386,000 per QALY gained. For individuals receiving cetuximab the expected cost per person for cetuximab was $33,360 and the expected incremental cost of cetuximab plus chemotherapy, relative to individuals who received chemotherapy only, was approximately $35,000 per person.

### Sensitivity analyses

The model was not sensitive to the disutility associated with severe AEs, the rates of AEs or the cost of severe AEs. The ICER remains above $200,000 per QALY when we changed these variables in one way, two way and three way sensitivity analyses. When we did not consider quality of life, cetuximab plus platinum-based chemotherapy compared to platinum-based chemotherapy alone led to an increase of 0.136 life years (LY) per person, resulting in an ICER of $265,000 per LY gained. When we used a time horizon of 2 years (the end of the follow-up period in the EXTREME trial), the ICER fell slightly to $340,700 per QALY gained. When we extended the time horizon to 4 and 5 years, our base case ICER estimates rose slightly to $393,000 per QALY gained and $395,000 per QALY gained, respectively. In addition, our results in the base case analysis remained robust when we varied the discounting rate for future costs and utilities between 0 to 5%.

**Figure 2:**
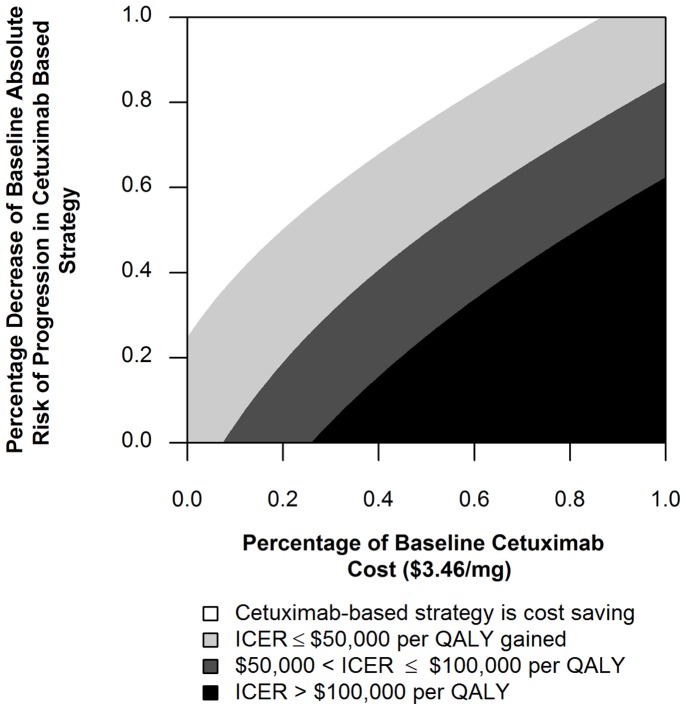
Sensitivity of the ICER to the cost of cetuximab per mg and the risk of progression in the cetuximab based strategy.

We conducted threshold analysis to identify conditions under which the ICER would fall below $100,000 per QALY gained. The ICER fell to less than $100,000 per QALY gained if the cost per mg of cetuximab was reduced by 75% to $0.81/mg (Figure 2). The ICER fell to less than $100,000 per QALY gained if the baseline absolute risk of progression in the cetuximab based strategy was reduced by 65% (Figure 2). In two way sensitivity analysis, the ICER fell to less than $100,000 per QALY gained, when simultaneously, the baseline cost per mg of cetuximab and risk of progression in the cetuximab based strategy were reduced by 40% and 35% respectively (Figure 2).

**Figure 3:**
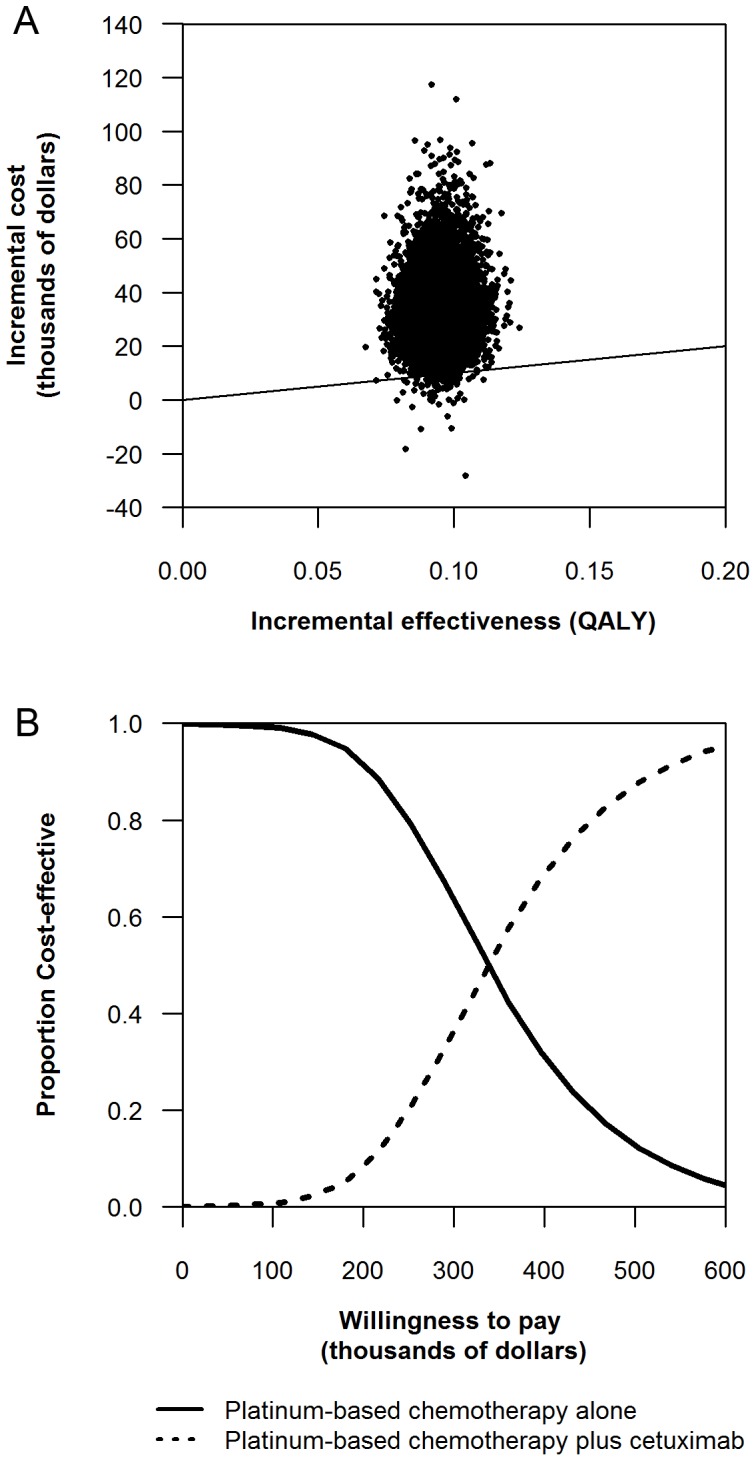
Incremental cost-effectiveness scatter plot and cost effectiveness acceptability curves of platinum-based chemotherapy plus cetuximab versus platinum-based chemotherapy alone. Each graph was based on 10000 replicates. 3a Incremental cost-effectiveness scatter plots. 3b Cost effectiveness acceptability curves.

We also performed a probabilistic sensitivity analysis and value-of-information analysis. We simultaneously varied all parameters (probabilities, utilities and costs) using appropriate distributions (Tables 1, 2, 3). Using a willingness to pay threshold of $100,000 per QALY gained, we found that the cetuximab based strategy was the preferred strategy in only 1% of simulations (Figure 3a). The cetuximab based strategy becomes equally favored at a willingness to pay of approximately $350,000 per QALY (Figure 3b). In addition, we performed value-of-information analysis [33]. Using a willingness to pay threshold of $100,000 per QALY gained, we found no value of removing all statistical uncertainty related to the benefit of cetuximab.

## Discussion

We developed a decision-analytic model to assess the cost effectiveness of cetuximab plus platinum-based chemotherapy versus platinum-based chemotherapy alone in first-line treatment of recurrent or metastatic HNSCC. In the base case, we estimated that cetuximab has an ICER of $386,000 per QALY gained. Our ICER estimate is significantly higher than $100,000 per QALY gained, a level which has been suggested in Canada to define “weak evidence in support of adoption” [34], and also above levels of recently rejected cancer treatments. However, funding decisions are not made solely on the basis of cost effectiveness, and other considerations such as need, equity and total budget impact may also be important to policy makers [35], [36], [37], [38].

Findings from the EXTREME trial indicate that adding cetuximab to platinum-based chemotherapy in first-line treatment of recurrent or metastatic HNSCC can lead to a modest but statistically significant and clinically meaningful survival benefit [8]. However, our analysis suggests that it may be challenging for public payers to fund cetuximab based on the current evidence.

Unlike cetuximab in the recurrent or metastatic HNSCC setting, the clinical effectiveness and cost-effectiveness of cetuximab has been previously demonstrated in locally advanced HNSCC [24]. Favorable ICER values were shown for patients who are medically unsuitable for concurrent platinum-based chemotherapy, with Karnofsky performance status (KPS) of 90% or better, or over the age of 70 years, with values ranging between €7,538 ($10,264 CAD) and €10,836 ($14,754 CAD) per QALY gained in Europe [24] and $19,740 CAD per QALY in Canada [39]. In those analyses, limiting cetuximab administration to patients who were most likely to benefit may have led to more favorable cost-effectiveness ratios. Consequently, cetuximab in combination with radiotherapy has been approved for reimbursement for these patient groups in the UK [10], [40] and Canada [41], [42]. More recent data suggest that no overall survival benefit is apparent in older patients, and these reimbursement decisions may warrant review [43].

Predictive biomarkers could improve cost effectiveness by selecting patients most likely to benefit from the addition of cetuximab to chemotherapy. This has been demonstrated in patients with other types of cancer such as breast [44] and colorectal cancer [45], [46]. For instance, Mittman et al [18] have shown that restricting cetuximab to advanced colorectal cancer patients with wild-type KRAS reduces the ICER of cetuximab over best supportive care alone from $199,742 CAD per QALY to $120,061CAD per QALY. Consequently, cetuximab has been approved for reimbursement for wild-type KRAS advanced colorectal cancer patients in Canada. Subgroup analyses in the EXTREME trial suggest that cetuximab plus platinum-based chemotherapy offered greater survival benefits to some subgroups than others [11]. Age less than 65 years, KPS of 80 or more, and primary tumour site other than hypopharynx appeared to be favorable for improved progression free survival and overall survival with cetuximab-based treatment. However sub-group treatment interaction tests identified only one significant interaction, which was between treatment and the primary tumour site (P = 0.03), and due to the lack of adjustment for multiple testing and the small numbers of patients in some of the subgroups, the authors were not able to state with certainty that some groups did not benefit from cetuximab or to suggest the degree of benefit from cetuximab across the studied subgroups [11].

Analysis of the EXTREME trial demonstrated that among patients receiving cetuximab plus platinum-based chemotherapy, the development of grade 1 or higher skin reactions at a given time was associated with a 23% reduction in the risk of death and a 20% reduction in the risk of progression, compared with patients not developing skin reactions by that time [8]. However, as every patient needs to be treated to determine skin reaction, this is an inefficient biomarker, and may simply be a pharmacodynamics biomarker of drug dose (i.e., less rash indicates the need for higher cetuximab dose). Profiling colorectal tumours for wild type versus mutated KRAS gene has been valuable for selecting patients who are unlikely to benefit with cetuximab or panitumumab [44], [45], [46]; however, these KRAS mutations are uncommon in HNSCC [47], [48]. EGFR gene copy number as determined by fluorescence in situ hybridization (FISH) does not appear to influence response to cetuximab in recurrent or metastatic HNSCC [8]. Therefore, there is no current evidence to suggest any particular clinical characteristic or biomarker is of practical use for tailoring treatment with cetuximab in patients with recurrent or metastatic HNSCC.

As such, our analysis assumed all patients with recurrent or metastatic disease are suitable candidates for the treatment with cetuximab. Our sensitivity analyses suggest that cetuximab is too expensive for its modest clinical benefits when added to platinum-based chemotherapy in this patient setting. Thus, only a reduction in the cost of cetuximab can lead to favorable cost effectiveness ratios at the present time. Results of value-of-information analysis indicated that future research on cetuximab in the recurrent or metastatic setting where all patients are considered suitable candidates (i.e., trials in which there are no adequate selection criterion) for the treatment with cetuximab such as the EXTREME study may not have a large societal impact, especially when willingness to pay levels of recently accepted cancer treatments are considered. Thus, the identification of predictive markers to better define subgroups of patients with recurrent or metastatic HNSCC for whom cetuximab plus platinum-based chemotherapy may offer either greater or less survival benefits than others should be a priority.

Our analysis has limitations. The health effects data used in this economic evaluation were generated from a single clinical trial which may not reflect the experience of broader population of patients with recurrent or metastatic HNSCC in Canada. However, only one randomized phase III trial exists [8]. As most cancer treatments are less effective and more toxic when generalized to clinical practice, our ICER estimate likely represents a “best case” scenario. There also may be uncertainty around the utility values used in our model. NICE considered the quality of life collected and reported in the EXTREME trial as limited. This could have an effect on our estimated ICERs but varying these values in sensitivity analyses had minimal effects.

### Conclusion

In the base case, the ICER of cetuximab exceeded $100,000 per QALY gained. Compared with other possible uses of public health care funds in Canada, the addition of cetuximab to platinum-based chemotherapy does not appear to provide good value for money in first-line treatment of patients with recurrent or metastatic HNSCC. However, cetuximab could be economically attractive in this patient population if its cost was reduced by at least 75% or if predictive biomarker were identified that could limit the use of cetuximab to those who are expected to most likely benefit.
